# Development of an indirect enzyme-linked immunosorbent assay based on the nucleocapsid protein of bovine parainfluenza virus type 3

**DOI:** 10.3389/fcimb.2026.1822931

**Published:** 2026-04-22

**Authors:** Juan Liao, Xue Yang, Ruiqi Li, Aoyuntuya Zhang, Yu Han, Shanhui Ren, Shijun Bao, Jinxin Xie

**Affiliations:** 1 College of Veterinary Medicine, Gansu Agricultural University, Lanzhou, Gansu, China; 2 College of Life Science, Leshan Normal University, Leshan, Sichuan, China; 3 College of Veterinary Medicine, Xinjiang Agricultural University, Urumqi, Xinjiang, China; 4 Alxa Left Banner Livestock Improvement Station, Alxa League, Inner Mongolia, China; 5 College of Advanced Agricultural Sciences, Yulin University, Yulin, Shaanxi, China; 6 Lanzhou Veterinary Research Institute, Chinese Academy of Agricultural Sciences, Lanzhou, Gansu, China

**Keywords:** bovine parainfluenza virus type 3, indirect enzyme-linked immunosorbent assay, nucleocapsid protein, polyclonal antibody, serological detection

## Abstract

**Purpose:**

Bovine parainfluenza virus type 3 (BPIV3) is an important pathogen in the bovine respiratory disease complex. The purpose of this study was to develop an efficient and rapid serological detection method, an indirect enzyme-linked immunosorbent assay (iELISA), for BPIV3.

**Methods:**

The full-length N gene of BPIV3 was amplified from reverse-transcribed BPIV3 cDNA and ligated into the pET-28a prokaryotic expression vector. The recombinant target N protein was correctly expressed. After purification, the target N protein was used to immunize BALB/c mice to prepare polyclonal antibodies. After experimental optimization, an iELISA for detecting antibodies against the BPIV3 N protein was established using purified protein and prepared polyclonal antibodies.

**Results:**

The recombinant N protein was efficiently expressed after induction with isopropyl β-D-1-thiogalactopyranoside at 32°C for 16 h. Immunization of BALB/c mice with the purified recombinant N protein elicited polyclonal antibodies that specifically reacted with BPIV3, with an antibody titer of 1:256000. The optimized iELISA conditions were as follows. Microtiter plates were coated with antigen at 5× 10^−2^ μg/mL, incubated overnight at 4 °C, and blocked with 1% bovine serum albumin for 2 h at 37 °C. Subsequently, plates were incubated with primary polyclonal antibodies at a working dilution of 1:8000 and horseradish peroxidase-conjugated secondary antibodies at a dilution of 1:8000 for 1 h at 37 °C. Finally, the colorimetric reaction was allowed to proceed for 10 min. Specificity tests showed that this method had no serological cross-reaction with other pathogens. Both intra-assay and inter-assay coefficients of variation were lower than 10%. Positive signals were still detected when the clinical-positive BPIV3 serum was diluted 1:6400. The concordance rate between this method and the virus neutralization test for clinical serum samples was 95.8%. A total of 176 cattle serum samples were tested using this iELISA method, yielding a serum antibody positivity rate of 94.9%.

**Conclusion:**

The N protein polyclonal antibody prepared in this study provides an important biological basis for research on the mechanism of BPIV3 infection. The establishment of an iELISA can enable the rapid screening of clinical samples, thereby providing reliable technical support for the epidemiological investigation of BPIV3.

## 1 Introduction

Bovine respiratory disease Complex (BRDC) is a common respiratory syndrome in cattle herds, caused by multiple pathogens, environmental stress, and management factors. It is considered one of the most severe and costly cattle diseases worldwide (Snowder et al., 2006). BRDC is characterized by coughing, difficulty breathing, fever, depression, and loss of appetite, which significantly affect cattle growth and development, reproductive performance, and economic returns in cattle farming (Zhou et al., 2025; Bell et al., 2021). Among the complex pathogen spectrum of BRDC, BPIV3 is one of the most critical primary pathogens. BPIV3 often co-infects with other pathogens, such as *Mycoplasma bovis* (M. bovis), bovine respiratory syncytial virus (BRSV), bovine viral diarrhea virus (BVDV), and bovine infectious rhinotracheitis virus (BIRV), further aggravating the disease process, increasing the difficulty of treatment, and raising the mortality rate (Zhou et al., 2025; Brown et al., 2025; Cusack et al., 2003; Kamdi et al., 2025).

Since its first isolation in the United States in 1959, BPIV3 infections have been reported in many countries worldwide, including Japan, Australia, Argentina, and Korea, making it a significant cause of respiratory disease in cattle herds globally and causing significant economic losses to the cattle industry each year (Resinger et al., 1959; Konishi et al., 2014; Horwood et al., 2008; Maidana et al., 2012; Oem et al., 2013). In 2008, BPIV3 was first isolated from nasal swabs of diseased cattle in China, and its complete genome was sequenced. Phylogenetic analysis indicated that this BPIV3 isolate represents a novel genotype, designated BPIV3 genotype C (BPIV3c) (Zhu et al., 2011). To date, three genotypes of BPIV3 have been identified: A (BPIV3a), B (BPIV3b), and C (BPIV3c), with genotype C predominating in China (Neill et al., 2015; Wen et al., 2012; Xu et al., 2024). In recent years, outbreaks and epidemics of BPIV3 have occurred in many major cattle-producing provinces, including Heilongjiang, Liaoning, Inner Mongolia, and Shandong (Zhou et al., 2023; Wen et al., 2012; Zhou et al., 2025; Zhu et al., 2011; Leal et al., 2019). In some areas, the antibody positivity rate has reached 77.6% (Wang et al., 2014). The frequent occurrence of the disease has severely restricted the scale of development and economic benefits of the cattle industry, becoming an important safety hazard affecting the safe production of livestock in China.

BPIV3 belongs to the genus Paramyxovirus in the family *Paramyxoviridae*. It is an enveloped, non-segmented, single-stranded, negative-sense RNA virus. Its genome is approximately 15,456 base pairs (bp) in length and encodes six structural proteins: nucleocapsid (N), phosphoprotein (P), matrix (M), fusion (F), hemagglutinin-neuraminidase (HN), and large polymerase (L) (Sakai et al., 1987; Suzu et al., 1987; Pelet et al., 1991; Bailly et al., 2000). Among these proteins, the BPIV3 N protein serves as the core component of the viral nucleocapsid and is highly conserved in the viral structure. N protein is highly antigenic and can elicit specific antibodies, making it an ideal target for viral typing, diagnosis, and serological testing (Sakai et al., 1987). Similarly, N protein-based serological assays have been widely established for other paramyxoviruses, including the Newcastle disease virus (NDV) and human parainfluenza virus 4 (HPIV4) (Domańska-Blicharz et al., 2019; Bulavaitė et al., 2017).

Antibody detection is a key method for epidemiological surveillance and assessment of BPIV3 infection status in cattle. Several BPIV3 antibody serum detection methods have been established and reported, including the virus neutralization test (VNT), indirect enzyme-linked immunosorbent assay (iELISA), and indirect immunofluorescence assay (IFA) (Trybała et al., 1989; Ren et al., 2015; Alkan et al., 2000). Among these methods, iELISA has been widely used for serological screening of animal diseases due to its rapidity, high sensitivity, and high throughput. Polyclonal antibody preparation technology is well-established and cost-effective, and the resulting antibodies can recognize multiple antigenic epitopes, making them suitable for developing iELISA detection methods. Here, the full-length N protein of BPIV3 was cloned, expressed in prokaryotic cells, and used to immunize mice to generate polyclonal antibodies against BPIV3 infection. Subsequently, we aimed to develop a sensitive and accurate iELISA for the rapid diagnosis of BPIV3 infection and to promote the healthy development of the cattle industry.

## 2 Materials and methods

### 2.1 Virus, cells, test animals, and serum samples

BPIV3 (BPIV3/SX/2021) (GenBank Accession: ON804787) was gifted by Dr. Yu Han. The cell lines, including MDBK and Vero, were obtained from the National Collection of Authenticated Cell Cultures. BALB/c mice were purchased from the Lanzhou Veterinary Research Institute, Chinese Academy of Agricultural Sciences. BPIV3 antibody-positive serum was obtained from cattle naturally infected with BPIV3, and inactivated positive sera of Peste des petits ruminants virus (PPRV), Lumpy skin disease virus (LSDV), Orf virus (ORFV), and Goatpox virus (GTPV) were provided by the Lanzhou Institute of Animal Science, Chinese Academy of Agricultural Sciences, Lanzhou, China. Clinical samples suspected to contain BPIV3 were collected from both smallholder and commercial farms across 11 counties in the Xinjiang Uygur Autonomous Region, China.

### 2.2 Construction of prokaryotic expression plasmid

Prokaryotic expression primers targeting the full-length N gene of BPIV3 were designed using Snapgene software based on the complete genome sequences of BPIV3(BPIV3/SX/2021) in GenBank and synthesized by Azenta Biotechnology Co., Ltd. (primer information is listed in **Table 1**). Total viral RNA from BPIV3 was extracted using an RNA extraction kit, and the RNA was reverse-transcribed into cDNA using a commercial reverse transcription kit. The N gene was amplified by PCR using BPIV3 cDNA as the template and pET28-BPIV3-N-For and pET28-BPIV3-N-Rev as forward and reverse primers, respectively.

**Table 1:** Primer sequences used in this study.

Primer name	Sequence (5’–3’)	Fragment size
*p*ET28-BPIV3-N-For	cagcaaatgggtcgcGGATCCGCCACCatgttgagtctgtttgatacattcagtgcacgcaggc	1626 bp
*p*ET28-BPIV3-N-Rev	cttgtcgacggagctcGAATTCccGTGGTGGTGGTGGTGGTGGCTTCCTCCTCCgttacttccgaatgcgctgaacaggtcatctatctc	

The PCR-amplified BPIV3 N gene was ligated into the prokaryotic expression vector pET28a, which had been double-digested with B*am*H I-HF and E*co*R I-HF. The ligation product was transformed into competent E. coli DH5α cells, which were then plated on kanamycin-containing 2-YT solid medium and incubated overnight at 37 °C. Single positive colonies were picked and cultured under shaking for 4 h. Recombinant plasmids verified as positive by colony PCR were subjected to scale-up culture. The recombinant plasmid DNA was extracted and sent to Sangon Biotech (Shanghai) Co., Ltd. for sequencing verification. The correctly sequenced prokaryotic expression recombinant plasmid was designated pET28a-BPIV3-N.

### 2.3 Expression and purification of recombinant N protein

The recombinant plasmid pET28a-BPIV3-N was transformed into competent Rosetta cells using standard protocols. Positive colonies were picked and inoculated into kanamycin-resistant 2 YT liquid medium, incubated at 37 °C with shaking at 220 rpm until the OD_600_ reached approximately 0.6, and then induced with 0.5 mM IPTG for 12 h. A 1 mL aliquot of the bacterial culture was collected by centrifugation, and the protein expression pattern was analyzed via sodium dodecyl sulfate-polyacrylamide gel electrophoresis (SDS-PAGE). To optimize the expression efficiency, different IPTG concentrations (0, 0.1, 0.5, and 1 mmol/L), induction temperatures (28, 32, and 37 °C), and induction durations (4, 8, 12, and 16 h) were tested. Bacterial cells subjected to these conditions were collected, and SDS-PAGE was performed to identify the optimal induction parameters that maximized recombinant protein expression.

The target BPIV3 N protein was expressed under optimal conditions and purified by denaturation of the inclusion bodies and subsequent refolding. First, a large volume of the induced bacterial culture was centrifuged to harvest the bacterial pellet, which was then washed with phosphate-buffered saline (PBS) and disrupted by ultrasonication. Next, the inclusion body pellet was solubilized in denaturation buffer, stirred overnight at 4 °C at 120 rpm, and centrifuged to collect the supernatant, which contained the denatured inclusion bodies. Finally, the denatured inclusion bodies were placed in a dialysis bag and dialyzed sequentially against refolding buffers containing 6, 4, 2, and 0 mol/L urea. The supernatant was collected via centrifugation to obtain the purified protein. The purification efficiency of the target protein was assessed by Western blot, and the concentration of the purified protein solution was determined using a BCA protein assay kit.

### 2.4 Preparation and verification of the polyclonal antibodies targeting the BPIV3 N protein

To prepare polyclonal antibody targeting the BPIV3 N protein, the purified recombinant protein was mixed with Freund’s complete adjuvant at a 1:1 (v/v) ratio, and the mixture was fully emulsified until a stable water-in-oil emulsion was formed. Healthy 8-week-old male BALB/c mice were selected as immunized animals, and each mouse was subcutaneously immunized with the emulsified mixture at a dose of 250 μg recombinant BPIV3 N protein. A total of three immunizations were administered, with an interval of fourteen days between each. Specifically, the first immunization was conducted with the recombinant BPIV3 N protein mixed with Freund’s complete adjuvant, while the second and third immunizations were performed with the recombinant BPIV3 N protein mixed with Freund’s incomplete adjuvant at a 1:1 v/v ratio to enhance the specific immune response of the mice to the BPIV3 N protein. Fourteen days after the third immunization, blood samples were collected from the tail tip to detect polyclonal antibody production. Once the polyclonal antibody titer reached the standard, blood was collected via enucleation, and the serum was separated to obtain the target polyclonal antibodies, and their titers were determined by Western blot.

The eukaryotic expression plasmids (pCAGGS-BPIV3-N-Flag and the empty vector pCAGGS) were transfected into Vero cells. Meanwhile, Vero cells were infected with BPIV3. The prepared polyclonal antibodies and Flag-tagged antibodies were used as primary antibodies, and Alexa Fluor 488-conjugated goat anti-rabbit IgG (H+L) or Alexa Fluor 488-conjugated goat anti-mouse IgG (H+L) were used as secondary antibodies to verify the specificity of the polyclonal antibodies via IFA.

### 2.5 Establishment of the indirect enzyme-linked immunosorbent assay

Checkerboard titrations were performed to determine the optimal antigen coating and enzyme-labeled secondary antibody concentrations. The purified recombinant N protein was serially diluted in 0.01 mol/L PBS (pH 7.4) to final concentrations of 5, 5× 10^−1^, 5× 10^−2^, 5× 10^−3^, 5× 10^−4^, and 5× 10^−4^ μg/mL. Aliquots of 100 μL per well of the diluted protein were added to a 96-well microplate, which was then coated overnight at 4 °C. After three washes with PBST, the microplate was blocked with 1% BSA at 37 °C for 1 h, followed by three additional washes with PBST.

Polyclonal antibody serum and negative serum, both diluted 1:1000 in PBS, were added to the wells and incubated at 37 °C for 1 h, followed by three washes with PBST. Subsequently, enzyme-labeled secondary antibodies at dilutions of 1:2000, 1:3000, 1:4000, 1:5000, 1:6000, 1:7000, and 1:8000 were sequentially added, incubated at 37 °C for 1 h, and washed 3 times with PBST. Thereafter, 100 μL of TMB substrate solution was added to each well, and the microplate was incubated at 37 °C in the dark for 15 min to develop the color. The reaction was terminated by adding 50 μL of 2 mol/L H_2_SO_4_ stop solution to each well. Optical density at 450 nm (OD_450_) was measured using a microplate reader. The combination of antigen-coating concentration and enzyme-labeled secondary antibody concentration that yielded the maximum P/N ratio (the OD_450_ values ratio of positive to negative sera) was defined as the optimal condition.

Using the optimal antigen coating concentration and enzyme-labeled secondary antibody concentration obtained in the previous step, we optimized the following reaction conditions: BPIV3 N protein polyclonal antibody dilution (1:2000, 1:4000, 1:8000, 1:16000, 1:32000, and 1:64000), coating conditions (37 °C for 1 h, 37 °C for 2 h, and 4 °C for 12 h), blocking fluid (1% BSA, 1% FBS, and 5% skimmed milk powder), blocking temperature and time (37 °C for 1 h, 37 °C for 2 h, and 4 °C for 12 h), enzyme-labeled antibody incubation time (37 °C for 30, 60, 90, and 120 min), and substrate incubation time (5, 10, 15, and 20 min).

### 2.6 Determination of the iELISA cut-off value

Based on the optimized experimental conditions, 30 negative serum samples were subjected to iELISA detection, with each sample assayed in triplicate. The average value ( $\bar{X}$) and standard deviation (SD) of the OD_450_ values for the samples were calculated. In accordance with statistical principles, the criteria were set as follows: a serum sample was considered positive when its OD_450_ value was ≥ $\bar{X}+3\times \text{SD}$, negative when its OD_450_ value was ≤ $\bar{X}+2\times \text{SD}$, and suspicious when its OD_450_ value was between $\bar{X}+2\times \text{SD}$ and $\bar{X}+3\times \text{SD}$.

### 2.7 Specificity, sensitivity, and repeatability tests of the iELISA

For specificity analysis, the iELISA method described above was used to measure the OD_450_ values of sera positive for PPRV, LSDV, ORFV, GTPV, and other pathogens. Bovine serum free of antibodies against the aforementioned pathogens was used as a negative control to verify whether the developed iELISA exhibited cross-reactivity with positive sera from other bovine pathogens.

For the sensitivity analysis, the BPIV3 antibody-positive serum was serially diluted to ratios of 1:100, 1:200, 1:400, 1:800, 1:1600, 1:3200, 1:6400, and 1:12800. The OD_450_ absorbance values for each dilution were determined using the established iELISA, with 5 replicates per dilution. BPIV3 antibody-negative serum was used as a control to evaluate the sensitivity of the established iELISA.

For repeatability analysis, samples with different titers were selected, and each sample was assayed in five replicate wells using the iELISA method established in this study. For the inter-assay repeatability test, the detection protocol was performed using ELISA plates coated with recombinant BPIV3 N protein from two different batches. All test results were statistically analyzed to calculate the coefficient of variation (CV).

### 2.8 Clinical sample detection and iELISA preliminary application

The iELISA method established in this study was used to detect BPIV3 antibodies in 176 bovine serum samples collected from different regions of Xinjiang Uygur Autonomous Region, and the positivity rate was statistically analyzed. In addition, 24 serum samples were randomly selected for verification using the neutralization test, and the concordance rates of the two detection methods were compared.

## 3 Results

### 3.1 Inductive expression and purification of the BPIV3 N recombinant protein

**Figure 1:**
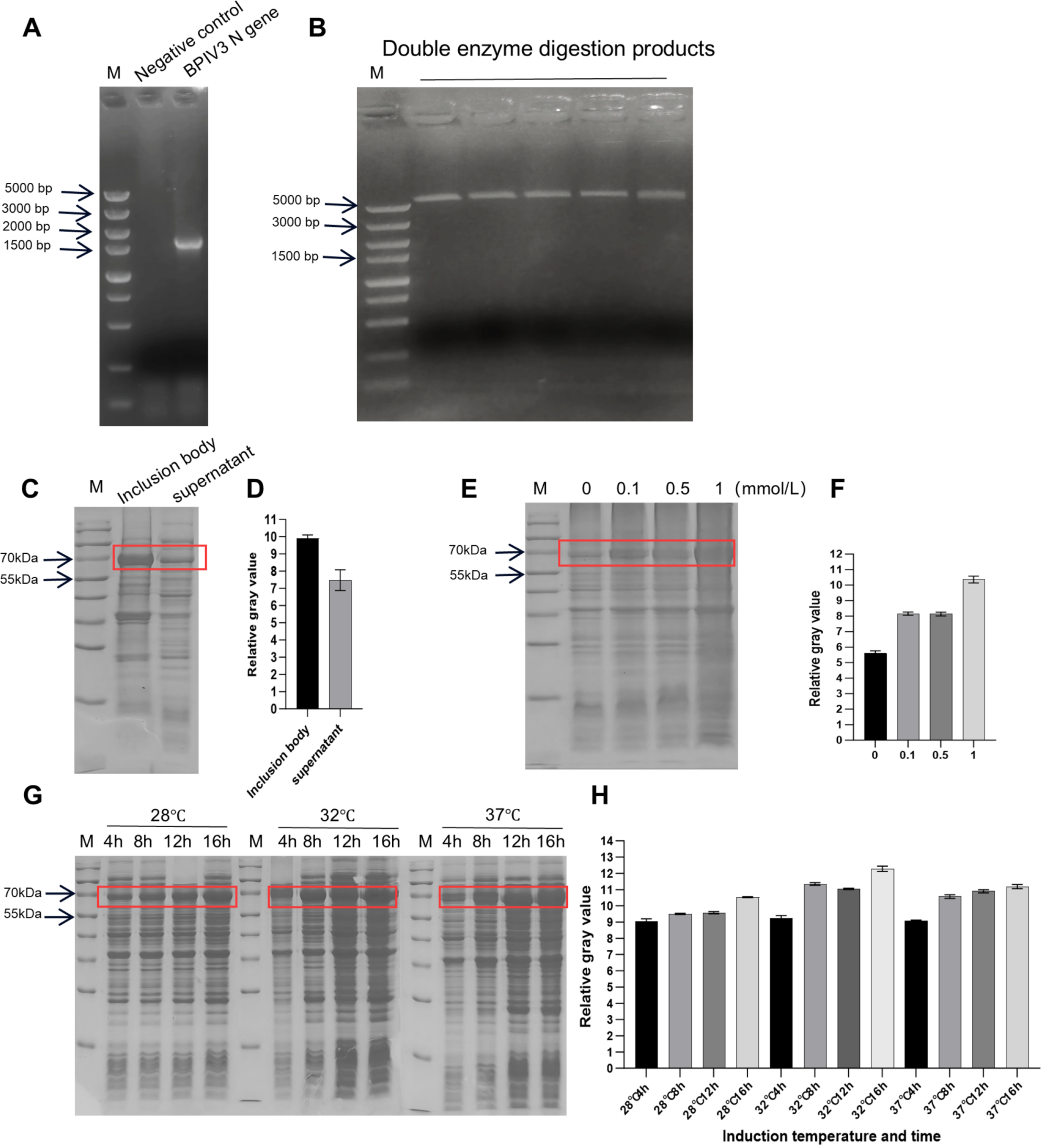
Construction of prokaryotic expression plasmid pET-28a vector and induced expression of BPIV3 N protein. **(A)** PCR results for the BPIV3 N gene. **(B)** Double digestion results of pET-28a with E*co*R I-HF and B*am*H I-HF. **(C)** Solubility analysis of the BPIV3 N recombinant protein. The recombinant bacterial cells were induced, harvested, and lysed by ultrasonic disruption, followed by centrifugation to separate the insoluble **(inclusion bodies)** and soluble fractions (supernatant). The distribution of the target protein between the two fractions was determined using SDS-PAGE. **(D)** Grayscale analysis of the target protein in inclusion bodies and supernatants. The gray value of the target protein band was quantified using ImageJ. **(E)** Concentration screening of the optimal induction agent IPTG for the BPIV3 N recombinant protein expression. Recombinant bacterial cells were induced with different IPTG concentrations (0, 0.1, 0.5, and 1 mM), and the expression level of the target protein was assessed using SDS-PAGE to determine the optimal inducer concentration. **(F)** Gray-scale analysis of target protein expression levels induced by different inducer concentrations. The gray value of the target protein band in each group was quantified using ImageJ. **(G)** Screening of the optimal induction temperature and time for BPIV3 N recombinant protein expression. **(H)** Gray-scale analysis of target protein expression levels at different induction temperatures and times. The gray value of the target protein band in each experimental group was quantified, and the relative expression levels were compared to confirm the optimal induction conditions.

The full-length BPIV3 N gene was amplified using the primers listed in **Table 1**. The results are shown in **Figures 1A, B**, with a band of approximately 1626 bp, which was consistent with expectations. The target fragment was ligated into the pET-28a vector, which was double-digested with E*co*R I-HF and B*am*H I-HF, via homologous recombination. The recombinant plasmid DNA after ligation was extracted. Sequence alignment analysis of the sequencing results demonstrated the successful construction of a prokaryotic expression plasmid for the BPIV3 N gene, designated pET28a-BPIV3-N.

To determine the expression form of the target BPIV3 N protein, the induced bacterial culture was disrupted by ultrasonication and centrifuged to collect the supernatant and inclusion body precipitate, which were then subjected to SDS-PAGE and grayscale analysis. The SDS-PAGE results are shown in **Figures 1C, D** showed that the BPIV3 N recombinant protein was expressed in both the supernatant and inclusion bodies, with the latter being the predominant form. To screen for the optimal IPTG concentration for target protein expression, IPTG was added at 0, 0.1, 0.5, and 1 mM, and the bacterial culture was induced at 37 °C for 12 h. The expression levels under these conditions were detected by SDS-PAGE and grayscale analysis, and the results are presented in **Figures 1E, F**. Expression of the target protein increased significantly after IPTG induction, with the highest level observed at 1 mM IPTG. To screen for the optimal induction temperature and time, 1 mM IPTG was used to induce at 28, 32, and 37 °C for 4, 8, 12, and 16 h, and the expression levels were analyzed by SDS-PAGE and grayscale analysis (**Figures 1G, H**). Based on the grayscale analysis shown in **Figure 1H**, the highest relative expression level of the target protein was observed with induction at 32 °C for 16 hours. Therefore, the optimal induction conditions for the expression of the BPIV3 N recombinant protein in this study were determined to be 1 mM IPTG at 32 °C for 16 h.

### 3.2 Preparation and verification of polyclonal antibodies targeting the denatured and native BPIV3 N protein

After large-scale expression of the target protein under optimal induction conditions, the bacterial pellet was washed with 0.01 mol/L PBS. The inclusion body precipitate was collected by centrifugation following ultrasonic disruption, washed, and denatured to obtain denatured inclusion bodies. The denatured inclusion bodies were refolded in buffers containing 6, 4, 2, and 0 mol/L urea, and the supernatant was collected by centrifugation to obtain the purified recombinant target protein. The concentration of the purified BPIV3 N protein was determined to be 1000.75 μg/mL using a BCA protein assay kit. Purified targeted N protein was used to immunize 20 healthy, 8-week-old male BALB/c mice. Fourteen days after the third immunization, tail-tip blood was collected for western blot analysis, and specific bands were detected in the sera of all 20 mice, indicating a 100% antibody detection rate. All mice underwent orbital blood collection to harvest serum.

**Figure 2:**
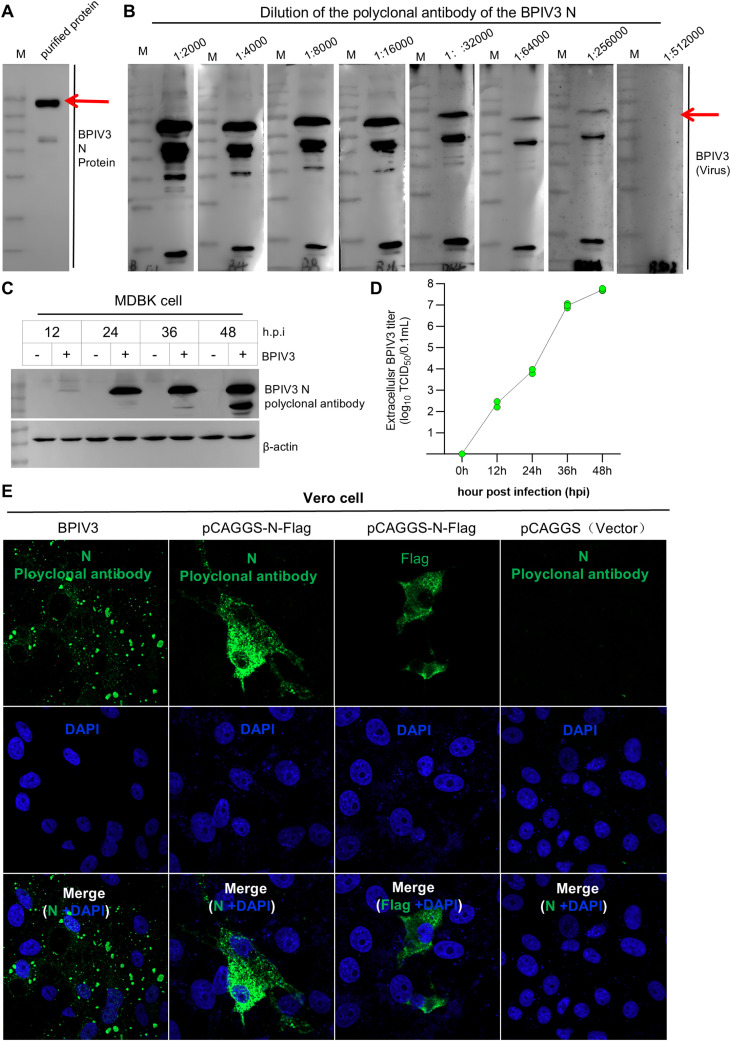
Purification of the target protein, viral titer determination, and verification of the polyclonal antibody against the BPIV3 N protein. **(A)** Western Blot analysis of purified BPIV3 N protein. **(B)** Western blot analysis of the titer of BPIV3 N protein polyclonal antibodies. Serial two-fold dilutions of the rabbit anti-BPIV3 N protein polyclonal antibody were used, ranging from 1:2000 to 1:512 000. **(C)** Western blot analysis of mock- and BPIV3-infected MDBK cell samples. MDBK cells were infected with BPIV3, and cell samples were collected 12, 24, 36, and 48 h post-infection. **(D)** TCID_50_ determination of mock- and BPIV3-infected MDBK cell supernatants. The TCID50 value was calculated using the Reed-Muench method, which reflects the viral titer in the infected cell supernatant samples. **(E)** IFA for detection of BPIV3 N protein expression. Cells were transfected with the eukaryotic expression plasmid pCAGGS-BPIV3-N-Flag as the experimental group, and empty vector pCAGGS as the negative control. For the positive control, cells were infected with live BPIV3 to mimic the natural expression of the N protein. IFA was performed using a polyclonal antibody against BPIV3 N protein.

To determine the titer of the prepared polyclonal antibody, the serum was serially diluted 2-fold, and the highest dilution at which it exhibited specific immunoreactivity with BPIV3 was determined by Western blot. The purified recombinant N protein was subjected to Western blot analysis. As shown in **Figure 2A**, the band size was consistent with the expected value, demonstrating the good reactivity of this polyclonal antibody with purification of the BPIV3 N protein. As shown in **Figure 2B**, a specific band was detectable at a serum dilution of 1:256,000, whereas no specific band was observed at a dilution of 1:512,000, demonstrating that the titer of the polyclonal antibody was at least 1:256,000 against BPIV3. To further identify the reactivity of the polyclonal antibody with intact BPIV3, a Western blot was performed to detect the N protein in BPIV3-infected MDBK cells. As shown in **Figure 2C**, the intracellular N protein level was time-dependently upregulated in BPIV3-infected MDBK cells at 12, 24, 36, and 48 h post-infection, and this trend was consistent with the TCID_50_ assay results for BPIV3-infected MDBK cells (**Figure 2D**). These findings demonstrate that the prepared polyclonal antibody specifically reacted with BPIV3 N and exhibited strong denatured antigen-antibody reactivity.

To further validate the specificity of the prepared polyclonal antibody, an indirect immunofluorescence assay was performed using an antibody against the BPIV3 N protein. As shown in **Figure 2E**, punctate green fluorescence was observed around the nuclei of Vero cells infected with BPIV3, and flocculent green fluorescence was detected around the nuclei of Vero cells transfected with the eukaryotic expression plasmid pCAGGS-N-Flag. In contrast, no fluorescent signal was detected in Vero cells transfected with the empty vector pCAGGS. These results indicate that the prepared polyclonal antibody specifically recognized the native BPIV3 N protein.

### 3.3 Establishment and optimization of the indirect enzyme-linked immunosorbent assay

To optimize the reaction conditions for the iELISA detection of the BPIV3 antibody, a checkerboard titration was performed. The highest positive/negative (P/N) value was used as the criterion for selecting the optimal protein-coating concentration and enzyme-labeled antibody dilution. As the results of P/N are shown in **Table 2**, the optimal protein coating concentration was 5×10^−2^ μg/mL, and the optimal dilution of the enzyme-labeled antibody was 1:8000.

**Table 2:** Determination of P/N values based on iELISA checkerboard titration for optimizing protein coating concentration and enzyme-labeled antibody dilution.

Enzyme antibody dilution	Antigen coating concentration (μg/mL)					
	5	5×10^-1^	5×10^-2^	5×10^-3^	5×10^-4^	5×10^-5^
1:1000	1.338	1.586	4.511	7.122	4.439	2.904
1:2000	1.713	2.006	5.391	7.131	4.439	2.167
1:3000	2.515	2.786	8.229	10.279	3.517	1.766
1:4000	3.406	3.694	10.982	12.274	3.984	2.399
1:5000	3.065	3.890	9.645	8.405	3.139	2.195
1:6000	5.408	9.025	15.538	4.813	2.131	2.513
1:7000	12.790	12.436	14.403	10.583	4.660	4.933
1:8000	14.610	13.495	**16.893**	6.171	2.621	3.781

The optimal reaction conditions were selected based on the maximum P/N ratio. Bold values represent the optimal antigen coating concentration and enzyme antibody dilution.

**Figure 3:**
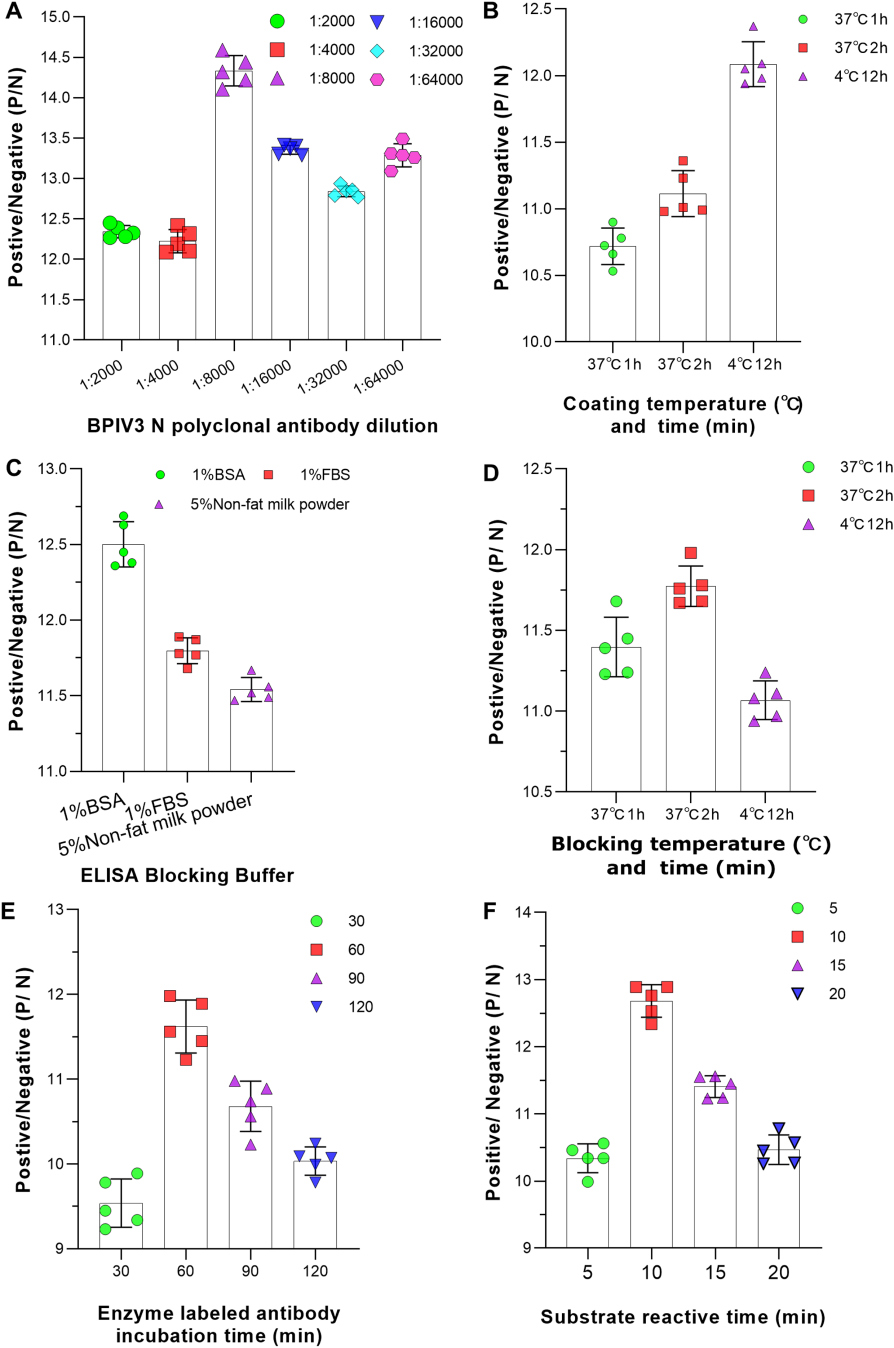
Optimization of the iELISA experimental conditions. **(A)** The optimal dilution of the polyclonal antibody was selected according to the positive/negative values. Different dilution gradients of the prepared polyclonal antibody were prepared, and the positive/negative (P/N) values for each gradient were measured using the established iELISA method. **(B)** The optimal conditions for the coating time/temperature were selected according to the positive/negative values. Different coating conditions were designed by combining temperature and the corresponding coating time. **(C)** The optimal blocking buffer was selected according to the positive/negative values. Different types of blocking buffers, including 1% bovine serum albumin (BSA), 1% fetal bovine serum (FBS), and 5% skimmed milk powder, were used to block the ELISA plates after antigen coating. **(D)** The optimal blocking time/temperature was selected according to the positive/negative values. Based on the optimal blocking buffer, different blocking times and temperature combinations were tested. **(E)** The optimal HRP-conjugated IgG incubation time was selected according to the positive/negative values. HRP-conjugated secondary antibody was incubated with the primary antibody-antigen complex at 37 °C for different times (30–120 min). The incubation time of the secondary antibody directly affects the signal intensity of the detection system. **(F)** The optimal chromogenic time was selected according to the positive/negative values. After adding the chromogenic substrate (TMB), the chromogenic reaction was carried out at 37 °C in the dark for different times (5, 10, 15, and 20 min).

Next, to establish the iELISA method, the remaining reaction conditions were optimized individually using a single-factor variable method, and the optimal settings for each parameter were determined. As shown in **Figure 3**, the optimal working dilution of the polyclonal antibody was 1:8000 (**Figure 3A**), the optimal protein-coating condition was incubation at 4°C for overnight (**Figure 3B**), the blocking condition was incubation with 1% BSA at 37°C for 2 h (**Figures 3C, D**), the incubation time for the enzyme-labeled antibody was 60 min (**Figure 3E**), and the optimal substrate reaction time was 10 min (**Figure 3F**).

### 3.4 Determination of the cut-off value and assessment of the specificity, sensitivity, and repeatability of the iELISA

**Figure 4:**
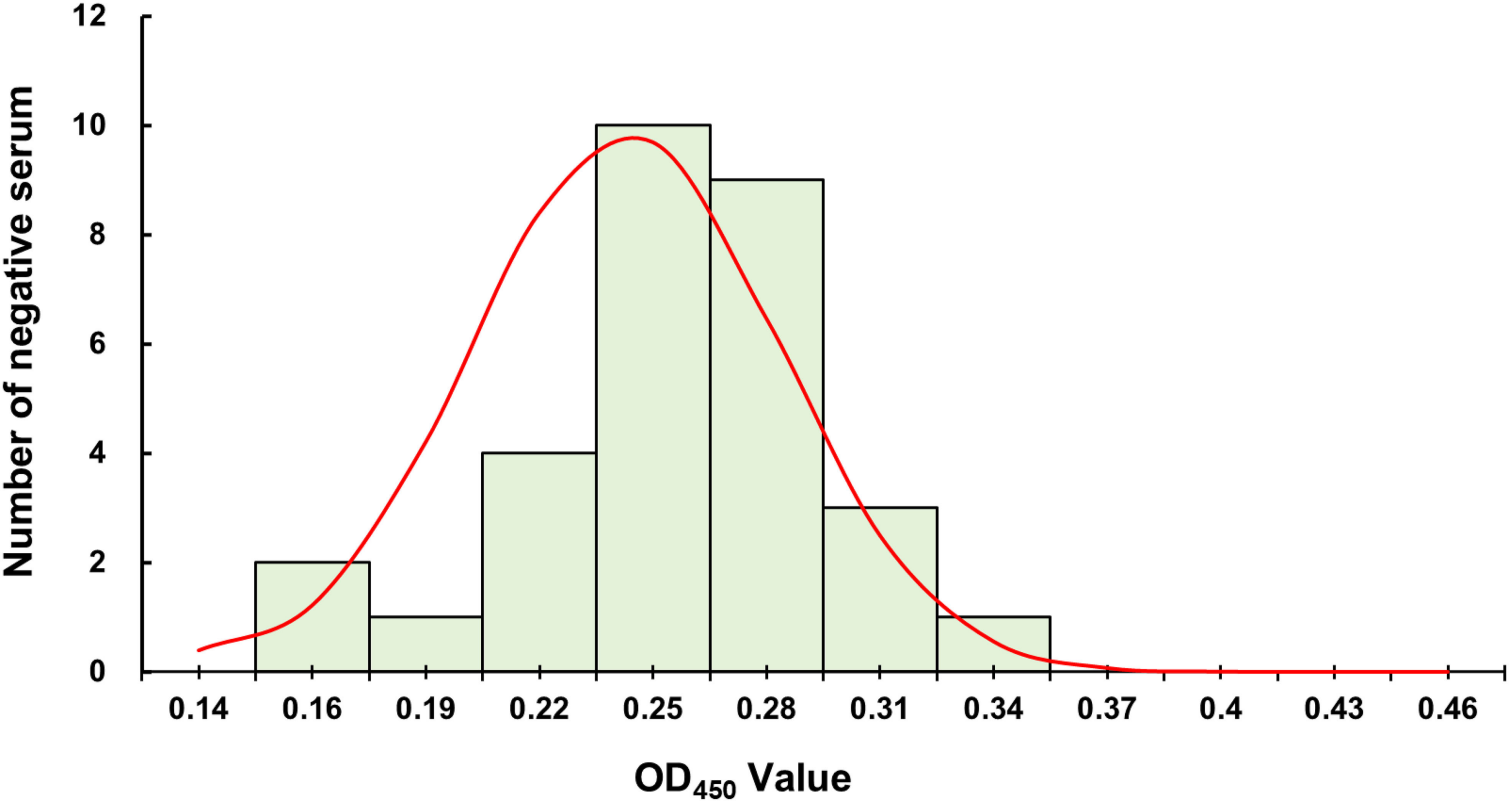
Normal distribution curve of OD_450_ values of negative serum samples. The curve illustrates the distribution characteristics of optical density values at 450 nm for negative reference sera, providing a basis for determining the cutoff value for subsequent serological assays.

To determine the cut-off value of the established iELISA method, thirty clinically negative serum samples were tested in triplicate, and the average value ( $\bar{X}$) and standard deviation (SD) of the OD_450_ values of the samples were calculated. The results showed that the serum OD_450_ values were normally distributed (**Figure 4**). After calculation, the mean was 0.240, and the standard deviation was 0.04. Based on this, the result determination criteria for this method were set as follows: OD_450_ ≥ 0.364 was judged as positive, OD_450_< 0.323 was judged as negative, and 0.323 ≤ OD_450_< 0.364 was judged as suspected.

**Figure 5:**
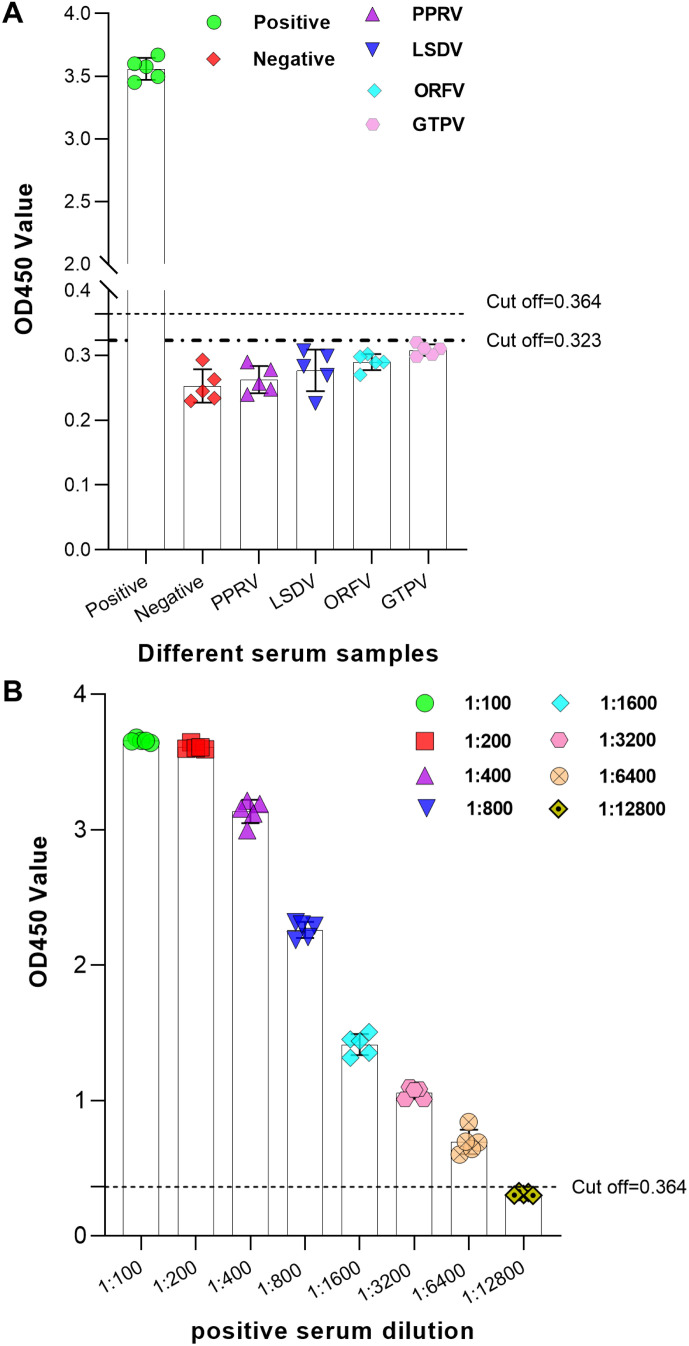
Specificity and Sensitivity Analysis of the iELISA. **(A)** Specificity analysis of the iELISA. Positive sera against PPRV, LSDV, ORFV, and GTPV were used as controls to evaluate iELISA specificity. **(B)** Sensitivity analysis of the iELISA. The BPIV3 antibody-positive serum was serially diluted to ratios of 1:100, 1:200, 1:400, 1:800, 1:1600, 1:3200, 1:6400, and 1:12800 for the sensitivity test.

To evaluate the specificity of the established iELISA method, positive sera against PPRV, LSDV, ORFV, and GTPV were employed as control samples and subjected to the same detection procedure. All OD_450_ values were below 0.323, indicating negative results for BPIV3 antibody detection (**Figure 5A**). These findings demonstrate that the established iELISA for BPIV3 antibody detection exhibits excellent specificity.

To evaluate the sensitivity of the established iELISA, BPIV3 antibody-positive serum was serially diluted from 1:100 to 1:12800, and the OD_450_ was determined for each dilution. The results showed that the positive serum was diluted 1:6400, and the detection result remained positive, indicating that the established iELISA exhibited good sensitivity (**Figure 5B**).

To verify the repeatability of the established iELISA method, both intra- and inter-assay repeatability were evaluated. Statistical analysis revealed that the intra- and inter-batch coefficients of variation (CVs) were both< 10%, indicating satisfactory assay reproducibility.

### 3.5 Clinical application and conformity verification of the iELISA

To comprehensively evaluate the clinical application potential and diagnostic coincidence rate of the established iELISA method, a total of 176 cattle serum samples were collected from multiple geographical regions across Xinjiang Uygur Autonomous Region, representing diverse breeding environments and cattle populations. These samples were subsequently analyzed using the iELISA established in this study. The results indicated that 167 of the 176 serum samples tested positive for BPIV3 antibodies, yielding an overall antibody-positive rate of 94.9% (167/176).

To further validate the reliability and accuracy of the established iELISA, 24 serum samples were randomly selected and subjected to a neutralization test, which is the gold standard method for BPIV3 antibody detection. Comparative analysis revealed that the two detection methods demonstrated a concordance rate of 95.8% (23/24). This high level of agreement strongly confirms that the iELISA method possesses satisfactory diagnostic specificity and consistency, making it a reliable tool for large-scale epidemiological investigations and for the clinical diagnosis of BPIV3 infection in cattle.

## 4 Discussion

This study focused on the highly conserved and immunogenic BPIV3 N protein, which was recombinantly expressed in a prokaryotic system and purified to obtain high-quality antigens. Immunization of BALB/c mice with the recombinant N protein elicited polyclonal antibodies with high specificity and high titers. An iELISA was subsequently developed and rigorously optimized for key parameters, including antigen coating concentration, blocking conditions, serum dilution, conjugate concentration, and substrate incubation time, to enable the sensitive and specific detection of BPIV3 specific antibodies. The established iELISA demonstrated excellent specificity, sensitivity, and reproducibility, with results closely correlating with those of the virus neutralization test. This study provides a practical and reliable tool for the clinical diagnosis, large-scale sero-surveillance, and epidemiological monitoring of BPIV3, as well as valuable antibody reagents for immunological studies.

Currently, the treatment of BPIV3 infection is mainly symptomatic and supportive, including antipyretics, anti-inflammatory agents, and replacement therapy, and there are no approved specific antiviral drugs (Makoschey and Berge, 2021). Rapid and accurate diagnosis is essential for preventing and controlling the transmission of BPIV3. Current antibody detection methods are primarily serological. Traditional serological detection methods include HA, HI, VNT, and IFA (Trybała et al., 1989; Werid et al., 2024; Trombetta et al., 2014). HA and HI assays detect antibodies by measuring the ability of BPIV3 to agglutinate chicken and guinea pig blood cells (Suzu et al., 1987; Trybała et al., 1989). The advantages of HA and HI are their low cost, simple operation, and quick results. However, the presence of nonspecific hemagglutination inhibitors, agglutinins, and endogenous interfering substances in serum samples can readily lead to false-positive or false-negative results, thereby limiting the accuracy of detection (Assaf et al., 1983). Moreover, hemagglutination activity is associated with the receptor-binding function of the viral HN protein, and if a viral strain undergoes a gene mutation or a decrease of hemagglutination during isolation and passage, this may directly lead to failure of the HA/HI method (Suzu et al., 1987). NT, a classic serological method, is highly accurate and is often used as a reference standard for novel detection methods. Moreover, VNT represents the most reliable surrogate of potency for passive immunotherapies, such as monoclonal or polyclonal antibody therapy (NT, a classic serological method, is highly accurate and is often used as a reference standard for novel detection methods. Moreover, VNT represents the most reliable surrogate of potency for passive immunotherapies, such as monoclonal or polyclonal antibody therapy (Focosi et al., 2021). However, the VNT method is cumbersome, with a long test cycle and the need to culture viruses and cells, making it difficult to meet the needs of rapid clinical diagnosis. However, the VNT method is cumbersome, with a long test cycle and the need to culture viruses and cells, making it difficult to meet the needs of rapid clinical diagnosis. Some researchers have developed an IFA for BPIV3 detection, which demonstrated high specificity and sensitivity and provided an effective means of detecting the BPIV3 antigen (Ray and Minnich, 1987). Nevertheless, this method requires advanced detection equipment and high operator technical proficiency, making it unsuitable for the rapid screening of large-scale samples.

The ELISA technique offers numerous advantages, including rapidity, cost-effectiveness, high-throughput capacity for large-scale sample processing, and ease of standardization (Yang et al., 2015; Florent and de Marneffe, 1986). In contrast to VNT, antibody-based ELISA does not require the handling of live viruses. Furthermore, ELISA can be used to determine antibody isotypes and subclasses (Graham et al., 1999). A comparative study of the sensitivity of ELISA and HI for antibody detection found that ELISA was 4–64 times more sensitive than HI (Assaf et al., 1983). Owing to these merits, ELISA has become one of the most widely employed and best-characterized serological methods for antibody detection in diverse viral infections (Baine et al., 2023; Wang et al., 2025; Afayibo et al., 2024). Researchers have significantly improved detection performance by optimizing the antigen and experimental design (Yang et al., 2015). However, existing ELISA kits have limitations, including incomplete coverage of antigen epitopes, low specificity, and cross-reactivity (Yang et al., 2015). Therefore, preparing polyclonal antibodies against the N protein with complete antigenic epitopes and establishing a novel ELISA detection method are important research directions to pursue. In this study, a novel iELISA method for detecting BPIV3 antibodies was established by optimizing key parameters, including coating antigen concentration, blocking conditions, serum dilution ratio, enzyme-labeled antibody concentration, and substrate reaction time. This method exhibited excellent sensitivity and specificity, with intra- and inter-assay CVs below 10%, thereby meeting the technical requirements for serological detection. The concordance rate between this method and VNT for detecting clinical serum samples was 95.8%, indicating its good practical utility. Previous studies have shown that antibodies against the N protein not only specifically recognize BPIV3 but also cross-react with some members of phylogenetically related respiratory virus genera (Ren et al., 2015). However, this method exhibited high specificity, with no cross-reactivity with common cattle and sheep pathogens, including PPRV, LSDV, ORFV, and GTPV.

Notably, the BPIV3 N protein is highly conserved across its three epidemic genotypes (A, B, and C) (Ren et al., 2015). Previous studies have confirmed that the BPIV3 N protein harbors highly conserved immunodominant antigenic epitopes that enable specific binding to BPIV3 antibodies without cross-reactivity with heterologous pathogens, thereby providing a solid molecular basis for the high specificity of this detection method (Sakai et al., 1987; Ren et al., 2015). Consistent with previous studies on BPIV3 serological detection, which also demonstrated no cross-reactivity with major related ruminant pathogens, we speculate that this is mainly attributable to the relatively conserved gene sequence and species specificity of the BPIV3 N protein (Yang et al., 2015). The titer of the prepared polyclonal antibody reached 1:256,000, laying a solid foundation for establishing a high-sensitivity detection system. Consequently, this method lowers the detection limit of positive sera to a high dilution of 1:6400, which is superior to some previously reported indirect ELISA methods and enables the reliable detection of samples with low antibody titers (Chen et al., 2021; Hou et al., 2022). This high sensitivity is particularly crucial for monitoring during early infection or when antibody levels are declining, as it can capture subtle changes that may be missed by low-sensitivity methods. The high sensitivity of this method is closely related to the high titer of the prepared polyclonal antibody and the high immunogenicity of the target antigen, which can effectively improve the detection rate of early- or sub-clinically infected samples. Additionally, this iELISA specifically targets BPIV3, including the main epidemic genotype C strain in China. It can be used for the clinical diagnosis and epidemiological investigation of BPIV3 and is of great significance for formulating targeted prevention and control strategies.

Although a previous epidemiological survey in China reported a lower BPIV3 positive rate in calves, the extremely high positive rate in this study suggests that BPIV3 may cause widespread subclinical or historical infections in cattle herds in the surveyed area (Ren et al., 2023; Xu et al., 2024). As reported in relevant reviews, subclinical BPIV3 infections are an important mechanism for the persistence and spread of the virus in cattle populations, suggesting that its epidemiological role in BRDC may be underestimated (Makoschey and Berge, 2021). Compared with the traditional virus neutralization test, the iELISA method established in this study has significant advantages, including simple operation, rapid detection, and suitability for high-throughput screening, making it more applicable for large-scale epidemiological investigations of clinical samples than the traditional test. In this study, screening of 176 serum samples from Xinjiang Uygur Autonomous Region using the iELISA method revealed a BPIV3 antibody positivity rate of 94.9%. This finding underscores the need to strengthen routine monitoring and epidemiological investigations of BPIV3 to provide effective technical support for the prevention and control of BRDC in cattle. Admittedly, this study also has certain limitations. First, although the specificity of the established iELISA was evaluated using several related viruses (PPRV, LSDV, ORFV, and GTPV), other common viral pathogens associated with BRDC, such as BVDV, BIRV, and BRSV, were not included in the specificity panel. Including these clinically relevant viruses in the detection panel would further enhance the reliability and clinical relevance of the assay. Second, all clinical samples were collected in the Xinjiang Uygur Autonomous Region, and it remains to be verified whether the results represent the epidemiological situation in other regions of the country. In addition, this method is primarily used for antibody detection and cannot distinguish between vaccine-induced and naturally acquired antibodies; therefore, caution should be exercised when interpreting results in cattle farms with complex immune backgrounds. In the future, the sample size and geographical scope can be further expanded to conduct nationwide epidemiological investigations of BPIV3 using this method. Meanwhile, efforts can be made to explore the integration of this method with detection methods for other core pathogens of BRDC, thereby providing more comprehensive technical support and a data basis for formulating integrated prevention and control strategies for BRDC.

## 5 Conclusion

In this study, the N gene of BPIV3 was successfully cloned and expressed, yielding a high-purity recombinant N protein. A polyclonal antibody with a high titer (1:256,000) and excellent specificity was generated against this protein. Using the purified recombinant N protein as the antigen, an iELISA for detecting BPIV3 N antibodies was developed. This iELISA showed no cross-reactivity and high sensitivity to related pathogens, including PPRV, LSDV, ORFV, and GTPV. Both the intra-assay and inter-assay coefficients of variation were less than 10%, and the concordance rate between this method and the VNT for clinical serum samples was 95.8%. The BPIV3 antibody-positive rate of the 176 clinical bovine serum samples from Xinjiang Uygur Autonomous Region was 94.9%. In conclusion, the N protein and polyclonal antibody prepared in this study provide important materials for BPIV3-related studies. The establishment of iELISA, with the advantages of rapidity, specificity, and reliability, offers an effective technical tool for the clinical screening and epidemiological surveillance of BPIV3.

## Data Availability

The datasets presented in this study can be found in online repositories. The names of the repository/repositories and accession number(s) can be found below: https://www.ncbi.nlm.nih.gov/genbank/, ON804787.
